# Human Second Window Pre-Conditioning and Post-Conditioning by Nitrite Is Influenced by a Common Polymorphism in Mitochondrial Aldehyde Dehydrogenase

**DOI:** 10.1016/j.jacbts.2016.11.006

**Published:** 2017-02-15

**Authors:** Julian O.M. Ormerod, Jonathan D.W. Evans, Hussain Contractor, Matteo Beretta, Sayqa Arif, Bernadette O. Fernandez, Martin Feelisch, Bernd Mayer, Rajesh K. Kharbanda, Michael P. Frenneaux, Houman Ashrafian

**Affiliations:** a Oxford Heart Centre, Oxford University Hospitals, Oxford, United Kingdom; b Department of Medicine, University of Cambridge School of Clinical Medicine, Cambridge, United Kingdom; c Department of Cardiovascular Medicine, West Wing, John Radcliffe Hospital, University of Oxford, Oxford, United Kingdom; d Department of Pharmacology and Toxicology, Karl-Franzens-Universität, Graz, Austria; e Department of Cardiovascular Medicine, Medical School, University of Birmingham, Birmingham, United Kingdom; f Faculty of Medicine, Clinical and Experimental Sciences, University of Southampton, Southampton, United Kingdom; g Medical School, University of East Anglia, Norwich, United Kingdom

**Keywords:** cytoprotection, endothelium, nitric oxide, nitrite, reperfusion injury, Ach, acetylcholine, ALDH2, mitochondrial aldehyde dehydrogenase, FBF, forearm blood flow, FBF-R, forearm blood flow ratio, GTN, glyceryl trinitrate, IR, ischemia–reperfusion, RIPC, remote ischemic pre-conditioning

## Abstract

Pre-conditioning is an exciting physiological phenomenon that, despite great efforts, has so far resisted translation to mainstream clinical medicine. Many potential triggers (e.g., ischemia of the organ in question or a remote organ, many different drugs) have been investigated, but recent work has implicated activation of mitochondrial aldehyde dehydrogenase (ALDH2) as central to the process. A genetic polymorphism, known as ALDH2*2, is common worldwide (present in up to 40% of Han Chinese people) and produces a functionally different enzyme. The authors used a variety of protocols in the human ischemic forearm model, in participants with both enzyme types, to assess cytoprotection with low-dose sodium nitrite and attempt to further elucidate the role of ALDH2.

## Highlights

• Pre- (and peri-ischemia) conditioning is a potentially powerful protector against ischemia–reperfusion injury, and activation of ALDH2 may be a critical step.
• The authors demonstrate second-window pre-conditioning (i.e., with the stimulus 24 h before ischemia) with-low dose sodium nitrite in the vascular endothelium of healthy human volunteers.
• They go on to show that nitrite, administered during ischemia, also affords protection to vascular endothelium in participants with the common worldwide variant ALDH2*2 enzyme, but not in those with wild-type ALDH2, using this particular protocol.
• This surprising result shows the challenges of translation in this particular area and the critical importance of dose, location, and timing of the conditioning stimulus.

## Visual Abstract

**Figure:**
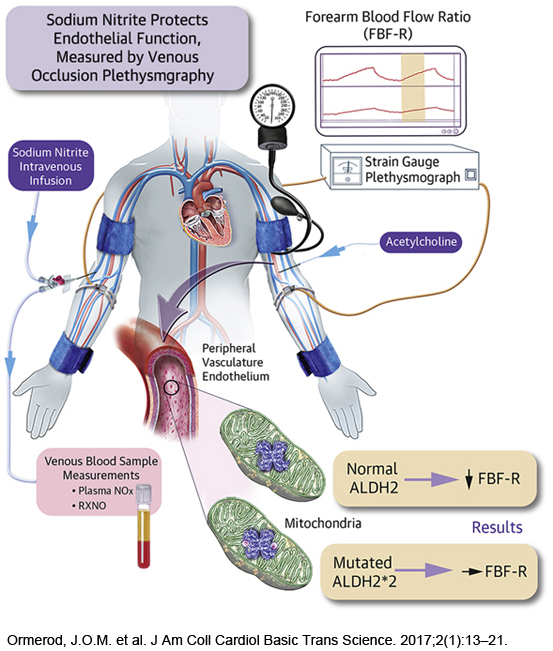

There has been great interest in possible beneficial effects of inorganic nitrite (NO_2_) in recent years 1, 2, 3. Nitrite may have a role in hypoxic vasodilation (4) and has been shown to protect renal tissue 5, 6, liver (7), and myocardium 8, 9 from ischemia–reperfusion (IR) injury in animal models. Nitrate (NO_3_^−^), administered orally in beetroot juice, protected endothelium from IR injury in healthy human volunteers (9), an effect attributed to a 2-fold increase in plasma nitrite. More recent work by Ingram et al. (10) in a human forearm model of reperfusion injury demonstrated a protective effect of a 20-min intravenous infusion of sodium nitrite (1.5 μmol/min) administered before the onset of ischemia (pre-conditioning), but administration of the same nitrite infusion during forearm ischemia (post-conditioning) resulted in no reduction in the degree of endothelial dysfunction compared with placebo. This lack of protection from nitrite administered during an ischemic insult contradicts the protective effects observed by Gonzalez et al. (8) in a canine model of myocardial infarction. The NIAMI (Nitrite in Acute Myocardial Infarction) study randomized 280 patients with ST-segment elevation myocardial infarction to receive 70 mmol sodium nitrite or placebo intravenously in the 5 min immediately before reperfusion (11). This post-conditioning protocol demonstrated no difference in infarct size at either 8 days or 6 months as determined by cardiac magnetic resonance imaging.

Mitochondrial aldehyde dehydrogenase (ALDH2) is a member of the 19-strong human aldehyde dehydrogenase family of NAD(P)^+^-dependent enzymes (12). A common polymorphism in exon 12 (Glu487Lys, or Glu504Lys in the unspliced protein), known as the ALDH2*2 allele, is present in up to 50% of individuals of East Asian descent (13). Heterozygosity at this allele results in a near inactive enzyme and produces the “Asian Flushing” phenotype, a phenomenon linked to the accumulation of acetaldehyde following alcohol ingestion; mutation of a single subunit destabilizes the cofactor binding site and dimer interface such that heterozygotes are functionally similar to homozygotes with the variant allele (14). Individuals possessing 1 or 2 copies of the ALDH2*2 allele may be at greater risk of coronary artery disease (15) and myocardial infarction (16). ALDH2 activation by phosphorylation has been postulated to be central to protection conferred against myocardial ischemia reperfusion injury (17). Pre-conditioning was induced by activation of PKCε (which phosphorylates ALDH2) and subsequently by a direct activator of ALDH2, alda-1. In a later study, the volatile anesthetic isoflurane induced cardioprotection in a rat model (18). Protection was associated with activation of ALDH2 and was abolished by an inhibitor of PKCε. On the basis of these data, combined with the observation that ALDH2 also exhibits intrinsic nitrite reductase activity (19), we hypothesized that an interaction between ALDH2 and nitrite might contribute to IR protection in humans.

We hypothesized that nitrite would be protective in the human forearm, either when administered 24 h before ischemia reperfusion (“second-window pre-conditioning”) or when administered during ischemia (with the primary effect in the “post-conditioning” window), and that its protective effects would be modified by variations in ALDH2 activity. We used a combination of genetic and pharmacological tools in an established model of IR injury in the human forearm (20), to investigate protection by nitrite and the role of ALDH2.

## Methods

This study was approved by the local research ethics committee. All participants gave written informed consent. All studies were performed in a dedicated vascular laboratory, maintained at 22°C to 24°C in quiet conditions. All participants were nonsmokers, and none was on medications of any kind. The baseline characteristics are given in Table 1. All participants avoided nitrite/nitrate-rich foods or alcohol for 24 h before the study, and abstained from caffeine on the day of the study. There was a washout period of >1 week between runs in the same patient.

**Table 1:** Baseline Characteristics of Participants in Each Group

	Delayed	*ALDH2∗1/∗2*	*ALDH2∗1/∗1*	Disulfiram
Height (m)	1.71 ± 0.05	1.72 ± 0.03	1.68 ± 0.03	1.83 ± 0.03
Weight (kg)	64.2 ± 6.3	61.6 ± 4.4	63.7 ± 2.6	77.5 ± 4.5
BMI (kg/m^2^)	21.5 ± 0.8	20.6 ± 0.8	22.6 ± 0.6	23.1 ± 1.0
Forearm (cm)	23.6 ± 0.9	23.0 ± 0.8	24.2 ± 0.4	25.6 ± 0.9
Age (yrs)	25.0 ± 1.0	23.0 ± 0.7	23.0 ± 0.7	24.0 ± 1.3
Individuals tested	7	11	11	6
Male/female	4/3	6/5	7/4	6/0
HR placebo (beats/min)	—	75 ± 3	61 ± 1	—
HR nitrite (beats/min)	66 ± 3	73 ± 3	64 ± 3	62 ± 5
MABP placebo (mm Hg)	—	84 ± 4	84 ± 5	—
MABP nitrite (mm Hg)	87 ± 4	85 ± 3	81 ± 3	89 ± 3

Values are mean ± SD or n.

ALDH2 = mitochondrial aldehyde dehydrogenase; BMI = body mass index; HR = heart rate; MABP = mean arterial blood pressure.

### Forearm venous occlusion plethysmography

Participants were seated upright on the bed with both arms exposed, fully extended, and supported at heart level. Mercury-in-silastic strain gauges of an appropriate size were placed around the widest portion of each forearm. The nondominant brachial artery was cannulated using a 27-gauge needle (Cooper’s Needleworks, Birmingham, United Kingdom) attached to an epidural catheter and sealed with dental wax under aseptic conditions, with lignocaine anesthesia at the participant’s discretion. Saline (0.9% NaCl, with or without agonists/drugs) was infused at 0.5 ml/min at all times to maintain needle patency. Venous access was gained with a 20-gauge cannula in the antecubital fossa of the control arm. Blood pressure and heart rate were continuously monitored. Forearm blood flow (FBF) was measured simultaneously in both arms using venous occlusion plethysmography to determine the FBF-ratio (FBF-R). Wrist cuffs were inflated to 200 mm Hg in order to exclude the hand circulation during measurements and were deflated between each set of measurements. FBF-R was measured at rest and again after 3-min intra-arterial infusion of acetylcholine (ACh) into the study arm at each of 3 ascending doses (25, 50, and 100 nmol/min). Endothelial function was assessed using the change in FBF-R from baseline. Endothelium-independent vasorelaxant effects were determined only once at the end of each study in the main group by intra-arterial infusion of glyceryl trinitrate (GTN) (at 3 ascending doses: 4, 8, and 16 nmol/min), in order to prevent potential confounding effects of GTN before ischemia (17).

### IR protocol

**Figure 1:**
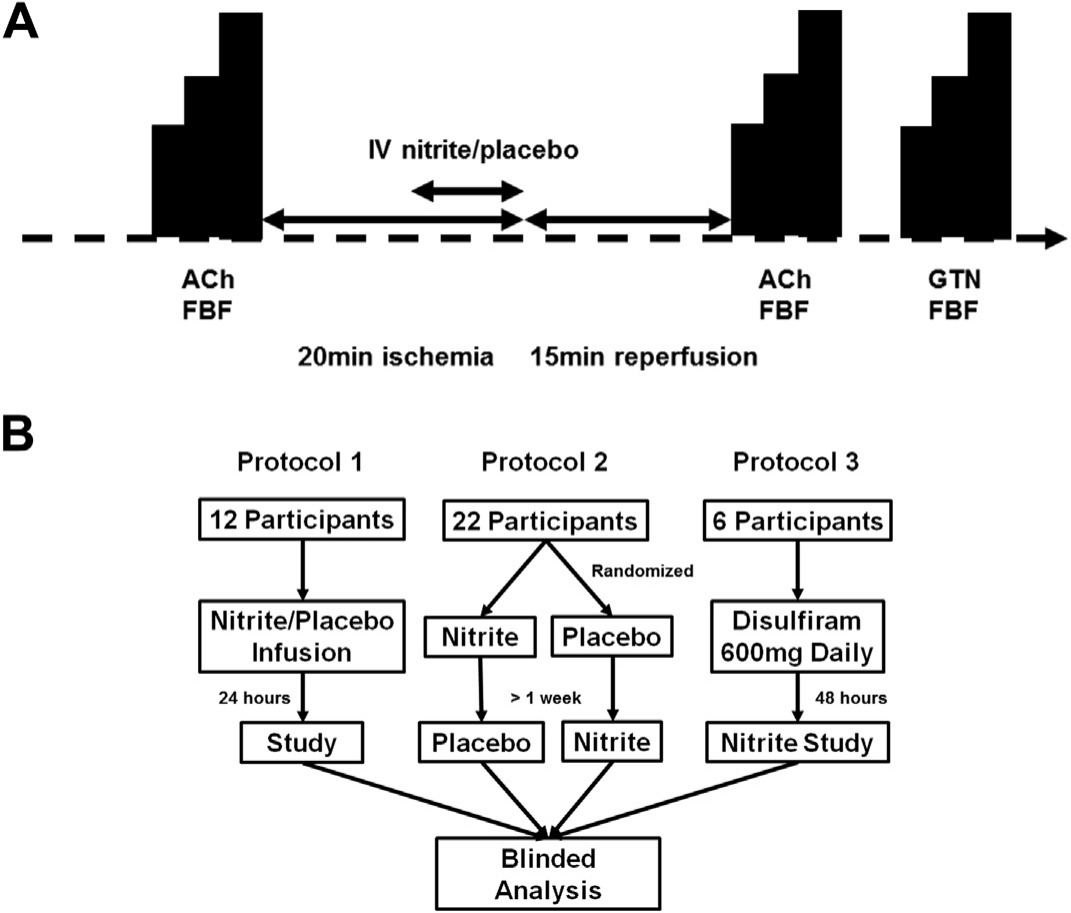
The Basic Study Design and Plan of Each Study **(A)** Basic design followed for each individual study, ACh FBF: measurement of endothelium-dependent forearm blood flow increase; GTN FBF: measurement of endothelium-independent forearm blood flow increase. **(B)** Summarizing the 3 protocols; there was a washout period of >1 week between any given studies in the same participant. Ach = acetylcholine; FBF = forearm blood flow; GTN = glyceryl trinitrate.

Following 15 min of stabilization following arterial cannulation, baseline endothelial function was measured. A 5-min washout was allowed to elapse from the end of the last infusion before forearm ischemia was induced by inflating the upper arm cuff on the study arm to >200 mm Hg for a period of 20 min. Endothelial function was then remeasured after 15 min of reperfusion, to assess the level of endothelial IR injury. This protocol has been demonstrated to cause transient endothelial dysfunction that is manifest after 15 min of reperfusion and lasting up to 60 min (20). The study design is summarized in Figure 1A.

### Nitrite infusion

Sodium nitrite (Martindale Pharma, Wooburn Green, United Kingdom) or saline placebo was infused intravenously into the control arm at a dose of 1 μg/kg/min for 10 min. The dosage of nitrite was chosen because it does not cause significant vasodilation or venodilation (4).

### Blood sampling

Venous blood samples were taken before ischemia and then after 1 min of reperfusion. Samples were placed in tubes spiked with EDTA (10 mmol/l) and NEM (2 mmol/l), transferred on ice to a chilled (4°C) centrifuge, and then spun at 4,000 rpm for 15 min. Plasma samples were then snap frozen in liquid nitrogen and stored at −80°C. Plasma nitrate/nitrite (NO_x_) and nitrosylated species (RXNO) levels were measured using gas-phase chemiluminescence (21).

### Determination of *ALDH2* genotype

Participants were not asked at any stage and were discouraged from discussing whether they possessed any features of the “flushing” phenotype in order to preserve blinding. Genetic analysis for *ALDH2* genotype was performed for all participants after the completion of studies, in order to maintain blinding. DNA was extracted from venous blood samples (QIAamp, Qiagen, Hilden, Germany) and analyzed using commercial probes and primers (Taqman System, Applied Biosciences, Thermo Fisher Scientific, Waltham, Massachusetts). One participant was found to be homozygous at the *2 allele. This dataset was excluded from the analysis.

### ALDH2-dependent inactivation by nitrite

Expression and purification of ALDH2 was performed as previously described (22). Dehydrogenase activity was measured by monitoring the formation of NADH as an increase in light absorbance at 340 nm in 50 mmol/l sodium pyrophosphate buffer (pH 7.5) containing 0.4 mmol/l acetaldehyde, 10 mmol/l MgCl_2_, and 5 mmol/l NAD. After 2 min of equilibration, the reactions were started by the addition of ALDH2*1 or ALDH2*2 (19 and 111 μg/ml final concentrations, respectively) and monitored for ∼3 min to obtain initial reaction rates. Activities were subsequently measured after the addition of 10 mmol/l NaNO_2_ and 1 mmol/l dithiothreitol.

### Treatment protocols

Treatment protocols are summarized in Figure 1B.

### Protocol 1: Second-window pre-conditioning by nitrite

Twelve Caucasians were recruited to the delayed (second-window) pre-conditioning group. A 10-min infusion of nitrite (or saline placebo) was administered intravenously 24 h before the onset of ischemia.

### Protocol 2: Effect of genetic polymorphism on first-window nitrite post-conditioning

Twenty-two healthy volunteers of East Asian origin were recruited to a randomized double-blind placebo-controlled crossover study. Nitrite or placebo was administered for 10 min during the second half of the 20-min ischemia period.

### Protocol 3: Effect of pharmacological inhibition of ALDH2 on first-window nitrite post-conditioning

Six Caucasians were pre-treated with disulfiram (600 mg/day) for 2 days before the study. In previous studies, this dose has been found to inhibit ALDH2 fully. Nitrite was administered for the latter 10 min of ischemia as in protocol 2.

### Statistical analysis

Data from each study were analyzed by an investigator who was blinded to the identity of the participants, their genetic status, and the protocol used. Statistical testing was performed using GraphPad Prism version 5.03 (GraphPad Software, La Jolla, California). Pre-and post-ischemia dose-response curves were compared using repeated measures 2-way analysis of variance. Other parametric values were compared with 2-way Student *t* test and nonparametric values with Mann-Whitney *U* test. A p value of <0.05 was considered statistically significant.

## Results

All subjects tolerated the procedures with no complications. Baseline characteristics of volunteers are given in Table 1. All Caucasian participants were homozygous for the wild-type ALDH2 (ALDH2*1/*1) allele. Of the East Asian participants, 11 were homozygous for wild-type ALDH2 (ALDH2*1/*1) and 11 were heterozygotes (ALDH2*1/*2). One was homozygous for the variant ALDH2 (ALDH2*2/*2) and was excluded from the analysis.

### Protocol 1: Second-window pre-conditioning by nitrite

**Figure 2:**
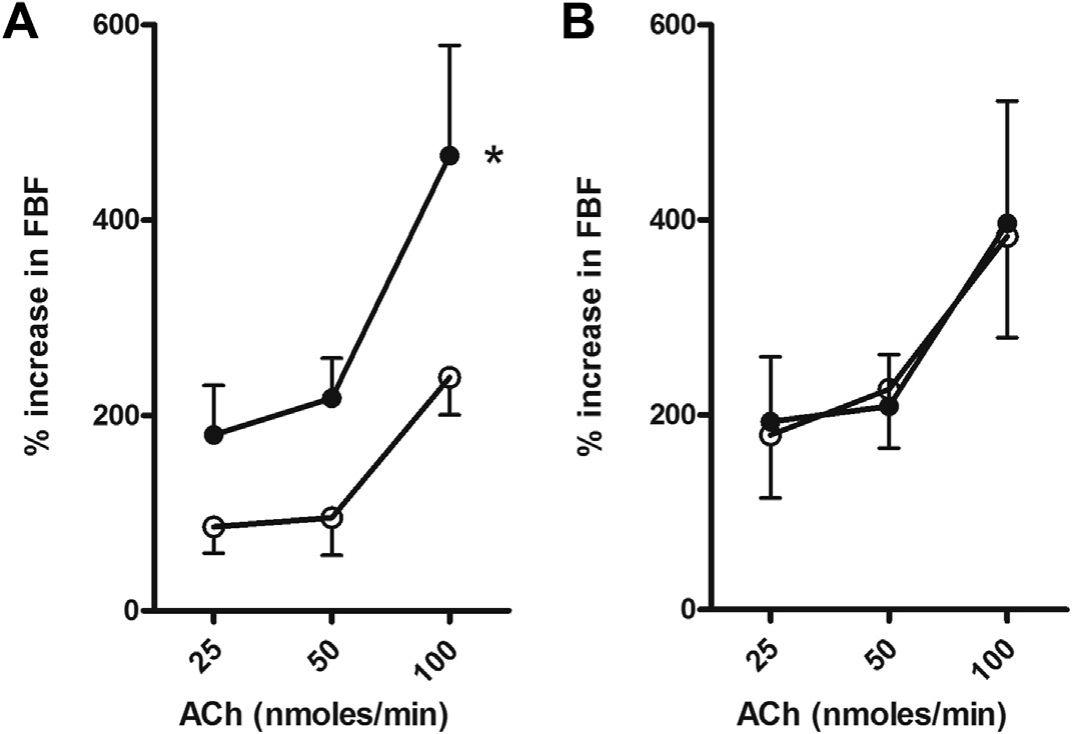
Intravenous Sodium Nitrite Induced Second-Window Pre-Conditioning in ALDH2*1/*1 Individuals Increase in FBF in response to ACh in individuals before and after 20 min of limb ischemia; **(A)** 20 min of forearm ischemia produced significant endothelial dysfunction (saline placebo 24 h pre-ischemia, pre- vs. post-ischemia FBF-R; p = 0.047 across all ACh doses, n = 5). **(B)** Intravenous sodium nitrite (1 μg/kg/min) administered 24 h before ischemia prevented endothelial dysfunction (pre- vs. post-ischemia FBF-R; p = 0.78 across all ACh doses, n = 7); *p < 0.05. **Solid circles** = pre-ischemia FBF-R; **open circles** = post-ischemia FBF-R. FBF-R = forearm blood flow ratio; other abbreviations as in Figure 1.

Twenty minutes of forearm ischemia followed by 15 min of reperfusion caused a reduction in FBF-R response to Ach, indicating endothelial dysfunction in those who received placebo infusion 24 h earlier (p = 0.047, n = 5). One μg/kg/min sodium nitrite administered intravenously 24 h before IR prevented this post-ischemia endothelial dysfunction in a group of homozygous wild-type (ALDH2*1/*1) participants (p = 0.78, n = 7) (Figures 2A and 2B).

### Protocol 2: Effect of genetic polymorphism on first-window nitrite post-conditioning

Sixteen of the 22 participants completed both studies, but the remaining 6 chose not to participate. As a result, 18 participants completed the placebo arm of the study (9 ALDH2*1/*1 and 9 ALDH2*1/*2), and 18 participants completed the nitrite arm (10 ALDH2*1/*1 and 8 ALDH2*1/*2), and thus paired analysis was not possible.

**Figure 3:**
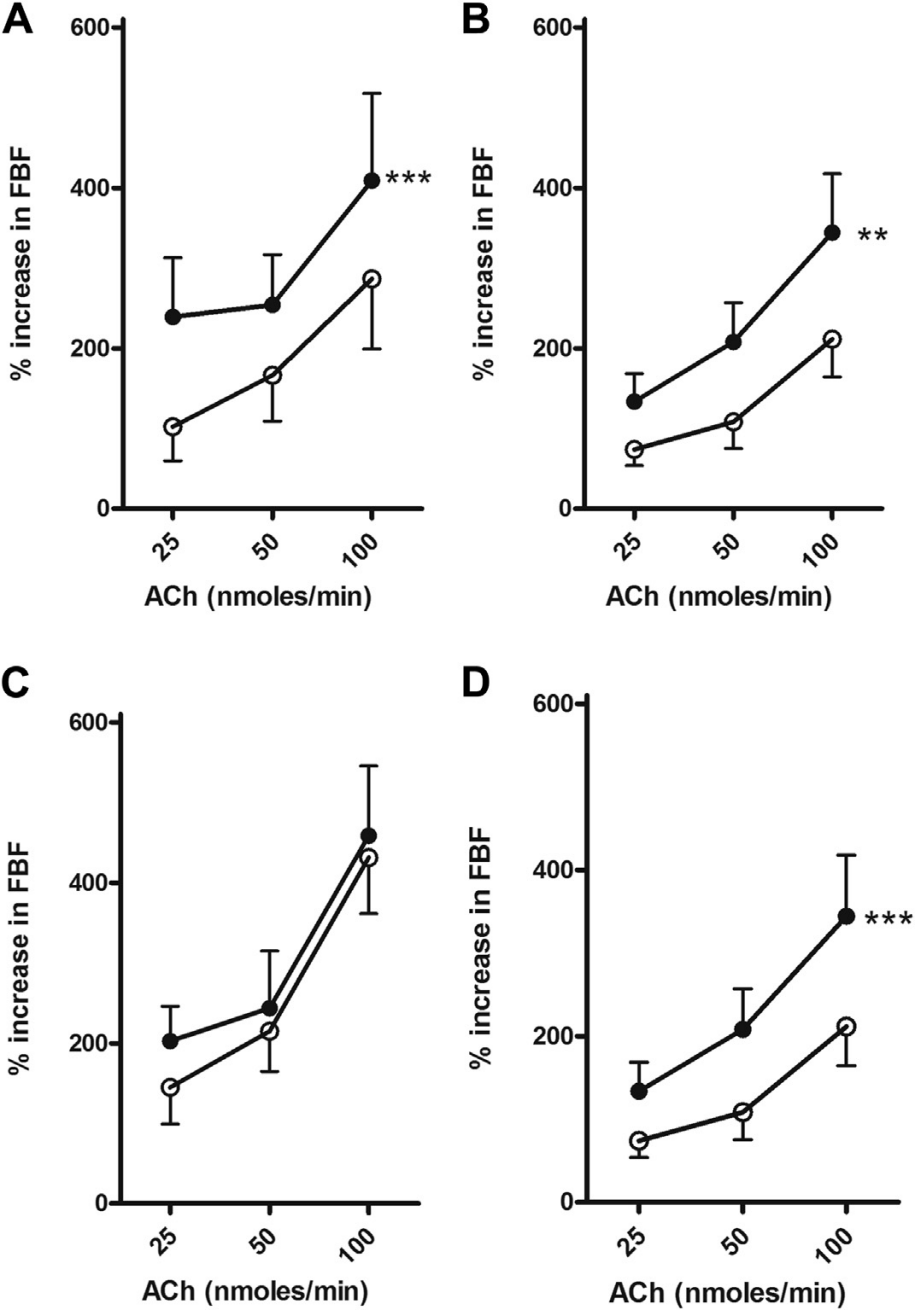
Intravenous Sodium Nitrite Induced Endothelial Post-Conditioning in ALDH2*1/*2 Individuals, But Not ALDH2*1/*1 Individuals **(A)** IR produced significant endothelial dysfunction in ALDH2*1/*2 individuals (p < 0.0001, n = 9). **(B)** IR produced significant endothelial dysfunction in ALDH2*1/*1 individuals (p = 0.0001, n = 9). **(C)** Intravenous sodium nitrite (1 μg/kg/min) administered during the final 10 min of ischemia prevented endothelial dysfunction in ALDH2*1/*2 individuals (p = 0.63, n = 8). **(D)** Intravenous sodium nitrite (1 μg/kg/min) administered during the final 10 min of ischemia did not prevent endothelial dysfunction in ALDH2*1/*1 individuals (p = 0.006, n = 10); **p < 0.01, ***p ≤ 0.0001. **Solid circles** = pre-ischemia FBF-R; **open circles** = post-ischemia FBF-R. IR = ischemia–reperfusion; other abbreviations as in Figures 1 and 2.

The IR protocol did not affect heart rate or blood pressure in any group; resting heart rate and blood pressure were similar on each study day (data not shown). Changes in endothelium-independent FBF were similar between groups (data not shown). Twenty minutes of forearm ischemia induced significant endothelial dysfunction in individuals with (p < 0.0001, n = 9) or without (p = 0.0001, n = 9) the ALDH2*2 allele (Figures 3A and 3B).

**Figure 4:**
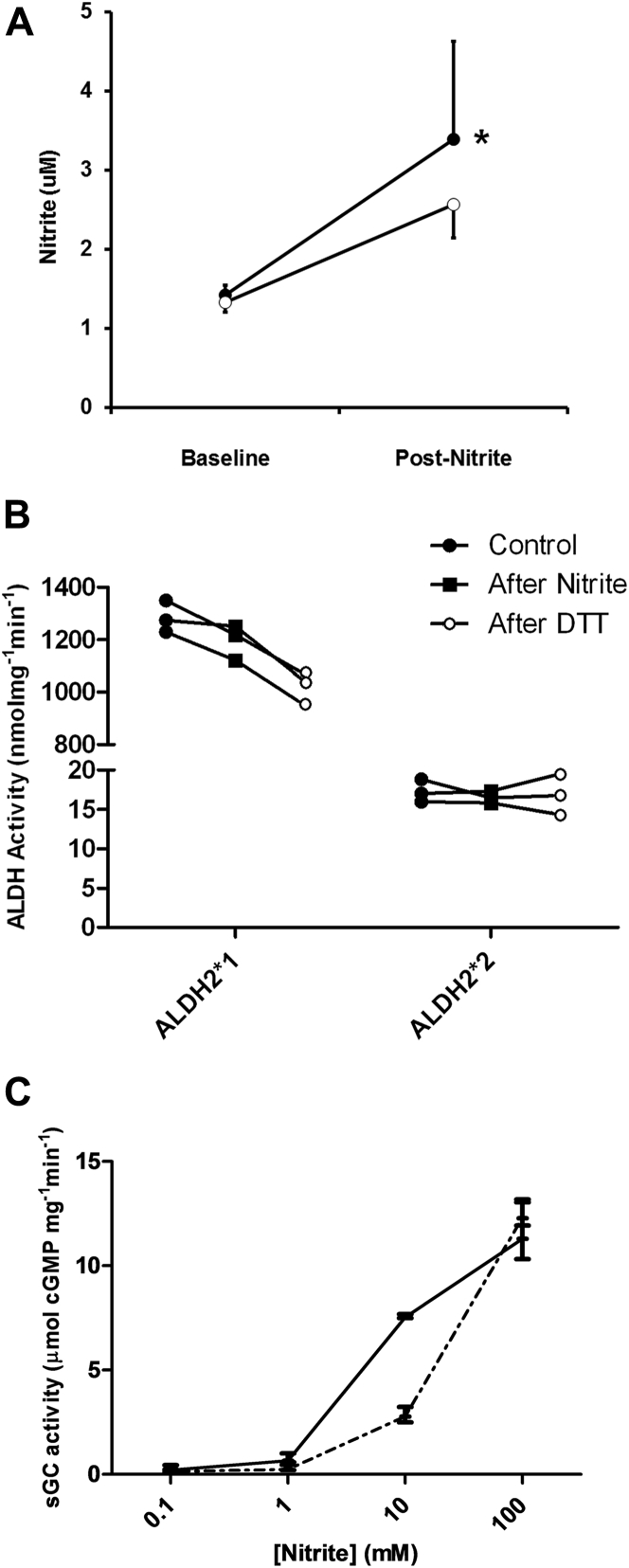
In Vitro Studies of Plasma Nitrite and Enzyme Activity **(A)** Plasma nitrite rose significantly in normal individuals, but did not reach significance in those with the *2 variant (*p < 0.05, n = 8 vs. 7). **Solid circles** = ALDH2*1/*1; **open circles** = ALDH2*1/*2. **(B)** Sodium nitrite inhibited aldehyde dehydrogenase activity of ALDH2*1 (p < 0.01 compared with baseline), but not *2, though the resting level of activity was far lower (n = 3 for each). **(C)** The ALDH2*2 variant enzyme exhibits a rightward shift in the NO-generation curve in response to nitrite when compared with the wild-type enzyme (p < 0.05, n = 3). **Solid line** = ALDH2*1; **dotted line** = ALDH2*2. DTT = dithiothreitol; sGC = soluble guanylate cyclase.

Plasma nitrite levels at baseline were similar in those with and without the ALDH2*2 allele. During studies where nitrite was administered, plasma nitrite increased from 1.43 ± 0.12 μmol/l to 3.39 ± 1.23 μmol/l in individuals with the *1 wild-type variant (p < 0.05, n = 8) but only from 1.33 ± 0.12 μmol/l to 2.56 ± 1.42 μmol/l in those with the *2 variant (Figure 4A). The plasma nitrate and RXNO were not significantly different at baseline and were not significantly different at 1 min of reperfusion (data not shown). Sodium nitrite mildly inhibited ALDH2*1 (by 6.4%; p = 0.04, n = 3), whereas ALDH2*2 was neither stimulated nor inhibited by nitrite (Figure 4B). The *2 variant enzyme exhibited a rightward shift in the soluble guanylate cyclase stimulation curve (a surrogate of NO generation) at moderate levels of nitrite (Figure 4C).

One μg/kg/min sodium nitrite administered intravenously during the latter 10 min of ischemia prevented endothelial dysfunction in participants that were heterozygous for the ALDH2*2 allele (p = 0.63, n = 8) (Figure 3C). Homozygous wild-type individuals did not display protection (p = 0.006, n = 10) (Figure 3D).

### Protocol 3: Effect of pharmacological inhibition of ALDH2 on first-window nitrite post-conditioning

**Figure 5:**
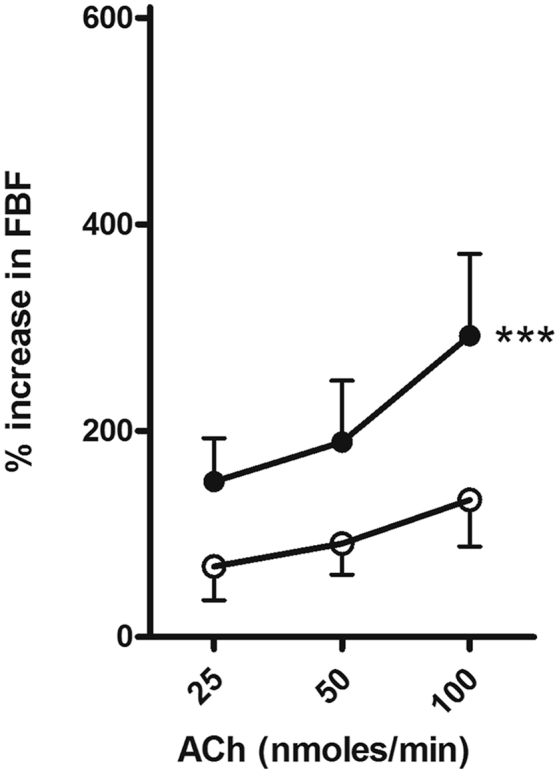
Disulfiram Pre-Treatment in ALDH2*1/*1 Individuals Did Not Recapitulate the Protection Seen in ALDH2*1/*2 Individuals Pre-treatment with disulfiram (600 mg daily for 2 days) did not rescue the wild-type phenotype (p < 0.0001, n = 6); ***p < 0.0001. **Solid circles** = pre-ischemia FBF-R; **open circles** = post-ischemia FBF-R. Abbreviations as in Figures 1, 2, and 3.

Homozygous wild-type participants pre-treated with disulfiram and administered 1 μg/kg/min sodium nitrite intravenously during the latter 10 min of ischemia displayed significant post-ischemic endothelial dysfunction (p < 0.0001, n = 6), suggesting no protective effect of nitrite in this setting (Figure 5).

## Discussion

We demonstrate for the first time to our knowledge in humans that nitrite administered 24 h before forearm ischemia attenuates the endothelial dysfunction following ischemia reperfusion injury. Furthermore, we show that nitrite post-conditioning is efficacious at reducing this dysfunction in certain individuals only, with protection depending upon *ALDH2* genotype. In order to establish whether reduced ALDH2 activity (known to be conferred by the mutation) was responsible for the observed effect, a group of homozygous participants were pre-treated with disulfiram. Pharmacological inhibition of ALDH2 in healthy volunteers did not recapitulate the ALDH2*1/*2 phenotype with respect to nitrite post-conditioning.

Second-window pharmacological pre-conditioning in humans has not been studied extensively. GTN, exercise, and remote ischemic pre-conditioning (RIPC) (where the stimulus for pre-conditioning is a different organ or tissue than the one protected, most commonly the lower limb in humans) have been studied in humans using angioplasty and experimental IR injury, but to our knowledge, our study is novel in demonstrating a late effect of nitrite pre-conditioning. This observation has implications for future studies to investigate the value of nitrite pre-conditioning in situations of predictable IR injury such as planned cardiac surgery and organ transplantation, and its additive role with first-window interventions.

The description of local post-conditioning has increased interest in recruiting these protective mechanisms in human IR syndromes where most patients present after the onset of ischemia. Thus, interventions timed at the phase of reperfusion may be clinically very relevant. Human forearm model data have suggested that pre-conditioning with nitrite may be effective, whereas they could not demonstrate an effect with post-conditioning (10). The NIAMI study in acute ST-segment elevation myocardial infarction was also negative (11). Our results in those homozygous for wild-type ALDH2*1 are consistent with these previous studies in demonstrating no reduction in ischemia reperfusion injury when nitrite is administered during ischemia. Interestingly, however, our data suggest that the post-conditioning effects of nitrite may dependent on *ALDH2* genotype. Thus, those individuals heterozygous for ALDH2*2, which confers its own functional biological effects (Figure 4), can be protected by nitrite at this time frame. Furthermore, simple pharmacological inhibition of the enzyme with disulfiram does not confer the same phenotype as in those with the mutation. This discrepancy in the effect of phenotypic and pharmacological inhibition of ALDH2 is in contrast to the observation that both prevent the protection conferred by RIPC (23), and is difficult to explain. It is possible that people with the ALDH2*2 gene change, who have abnormal enzyme function throughout life, possess compensatory adaptations elsewhere. Acute inhibition with disulfiram would not necessarily recapitulate these changes. The resulting enzyme created in those heterozygous for ALDH2*2 is not inert, and despite the vast number of people possessing this gene change worldwide, study of this enzyme has been limited. The 2 different enzyme forms directly, and most likely also indirectly, affect the metabolism and availability of nitrite and related species and this may well have contributed to the result observed.

### Study limitations

There are several limitations to the interpretation of this study: as with any pharmacological study, the dose of agent used may be inappropriate. The plasma nitrite concentration achieved in wild-type individuals in the present study is lower than that which successfully protected myocardium in animal models of pre-conditioning (cf. 13.8 μg/kg/min, 0.2 μmol/kg/min, aiming for a plasma concentration of 5 to 10 μmol/l in Gonzalez et al. [8]). However, the protective effect seen in those with the variant enzyme, who appear to have a smaller rise in plasma nitrite, may suggest that in the post-conditioning phase, this dose is too high. It has been reported that very high levels of NO may prevent protection (24). Thus, it is possible that, in the post-ischemia phase, the kinetics of nitrite metabolism are altered such that this dose is not protective. However, our inability to restore protection by pharmacological inhibition suggests that this enzyme system alone is not responsible for these effects. Although we cannot exclude off-target effects of disulfiram contributing to this observation, we believe that the interaction between nitrite and the ALDH2*1/*2 genotype is more nuanced than nitrite simply augmenting the detoxifying potential of an enzyme with reduced dehydrogenase activity (e.g., with respect to reactive aldehydes such as 4-hydroxy-2-nonenal). Supporting this inference, whereas our in vitro biochemical studies suggest that nitrite has little effect on ALDH activity per se, the differences in genotype nevertheless impact upon nitrite metabolism. It has been proposed that nitrite triggers a protective signaling cascade by reversible inhibition of mitochondrial complex I, with modulation of mitochondrial reactive oxygen species generation at reperfusion (25); however, the relevance of this mechanism in humans is unclear.

Because the study involves puncture of the brachial artery, it was felt that it was not reasonable to require more than 2 runs from each participant. Ideally, the effect of disulfiram and delayed protection would have been studied in the East Asian population, but insufficient participants were recruited. It was not possible to analyze as a crossover study, because not all participants completed both arms of the study. The data from placebo and nitrite runs were analyzed separately in the same manner as previously discussed 20, 26. There was a washout period of at least 1 week between studies that we believe was sufficient to preclude ongoing effects, and the placebo and nitrite studies were performed in random order. However, we cannot absolutely exclude effects of the previous study, and hence, we list this as a limitation. Finally, our disulfiram group consisted of men only, although there were no significant effects of sex found in any of the other groups. The biochemical study of ALDH2 inhibition/activation by nitrite was investigated using the homozygous variant enzyme because it is a dominant-negative polymorphic variant (22).

Clinical translation in the area of pre-conditioning and post-conditioning, despite ongoing efforts, has been slow. Direct activation of the ALDH2 pathway remains an attractive area of investigation (27); however, the negative NIAMI study (11) has somewhat reduced enthusiasm for further trials of exogenous nitrite in ischemia. More recent work has focused on a potential role of *endogenous* nitrite in cytoprotection, for example as a mediator of remote ischemic pre-conditioning (28). In a mouse model of myocardial infarction, this study confirmed the role of nitrite by use of a nitrite scavenger to abolish the effect of RIPC, and then recapitulated this protection with nitrite-supplemented plasma. In their editorial accompanying this study, Corti and Gladwin (29) argued that reliance on animal models may be limiting our ability to translate this undoubtedly exciting physiological process to mainstream medicine. Equally, human ischemic models (such as the one used here) may not translate to other tissues or organs, or to pathological states, though they may well provide insight into the mechanisms involved. It is generally agreed that cytoprotection proceeds down a final common pathway (30), so it seems likely that protection by nitrite seen in the forearm may be achieved in other tissues under the correct conditions. However, as Corti and Gladwin (29) argue, multiple factors—the nitrite dose, the plasma levels achieved, and the timing of intervention—create a complex system and are all critical to the successful translation of cytoprotection to human patients.

## Conclusions

This proof-of-concept study of endothelial protection in healthy volunteers provides evidence that inorganic nitrite may have therapeutic use to prevent IR injury in man. Specifically, we demonstrate nitrite-induced second-window pre-conditioning in humans and identify the important impact of a common global polymorphism on post-conditioning with nitrite.

Perspectives **COMPETENCY IN MEDICAL KNOWLEDGE:** IR injury is a common cause of cardiovascular injury or death. Pre-conditioning may reduce or prevent this injury in a variety of tissues, with effects over and above current treatments. Results from clinical trials thus far have been mixed, and translation to mainstream clinical medicine has been slow, despite increasing understanding of the relevant physiology. Here, we show a protective effect of low-dose systemic sodium nitrite in prevention of IR injury to the human vascular endothelium, when administered 24 h before the insult, and additionally in some participants (depending upon *ALDH2* genotype) when administered during ischemia.

**TRANSLATIONAL OUTLOOK:** Future translational studies should examine different protocols of nitrite dosage and timing in protection against IR injury. This work also sheds some additional light on the central, and complex, nature of ALDH2 in this process and complements recent work showing a potential role of nitrite as an innate mediator of remote ischemic pre-conditioning.
